# Pathological Vertebral Fractures Misdiagnosed as Osteoporotic Vertebral Fractures: A Case Series of Four Patients and Diagnostic Strategies

**DOI:** 10.1155/cro/7259158

**Published:** 2026-06-23

**Authors:** Jun-Ming Lin, Xiao-Jun Yuan, Guang Li, Xin-Rong Gan, Wen-Hua Xu

**Affiliations:** ^1^ Department of Orthopaedic Surgery, People′s Hospital of Yichun City, Yichun, Jiangxi Province, China

**Keywords:** brucellar spondylitis, multiple myeloma, osteoporotic vertebral fracture, pathological fracture, pyogenic spinal infection, tuberculous spondylitis

## Abstract

Nontraumatic vertebral fractures are common in the elderly, primarily caused by osteoporosis. However, pathological fractures (e.g., due to malignancy or infection) may exhibit clinical and radiological features similar to osteoporotic vertebral fractures (OVFs), leading to misdiagnosis. We report four cases of pathological vertebral fractures initially misdiagnosed as OVFs: one case of pyogenic spinal infection, one of tuberculous spondylitis, one of brucellar spondylitis, and one of multiple myeloma. These cases highlight the importance of considering further diagnostic evaluation, including biopsy, when laboratory abnormalities or atypical imaging features are present. Preoperative laboratory investigations, such as erythrocyte sedimentation rate (ESR), C‐reactive protein (CRP), albumin/globulin (A/G) ratio, and immunofixation electrophoresis, can provide critical clues for identifying underlying etiologies. Additionally, vertebral bone marrow aspiration biopsy during percutaneous vertebroplasty (PVP) is a simple and effective method to exclude hematological disorders and infection.

## 1. Introduction

Nontraumatic vertebral fractures in the elderly may arise from osteoporosis, infection, or malignancy. Osteoporotic vertebral fractures (OVFs) are the most common; however, pathological fractures caused by infection or hematological disorders can mimic OVFs in clinical presentation and radiological features, leading to misdiagnosis [1, 2]. Although established radiological criteria (e.g., magnetic resonance imaging (MRI)) exist to differentiate OVFs from pathological fractures, the absence of typical features in some cases increases diagnostic difficulty. Such misdiagnosis may delay appropriate treatment and adversely affect patient outcomes [3, 4].

This report presents four cases of pathological vertebral fractures (including infectious and malignant etiologies) initially misdiagnosed as OVFs (Table 1). Through these cases and a literature review, we aim to elucidate factors contributing to the misdiagnosis of pathological fractures as OVFs and propose strategies to enhance diagnostic accuracy.

**Table 1:** Baseline characteristics of the patients.

No	Age (years)	Sex	Chief complaint	Affected vertebra (e)	Intraoperative biopsy	Diagnosis
1	70	F	Low back pain	L3	Yes	Pyogenic spondylitis
2	89	F	Bilateral costal margin pain	T9,T10	Yes	Tuberculous spondylitis
3	74	F	Low back pain	L4	No	Brucellar spondylitis
4	70	F	Low back pain	L2	No	Multiple myeloma

## 2. Case Identification and Diagnostic Criteria

The four patients included in this case series were retrospectively identified from patients who presented to our department with symptomatic vertebral compression fractures between January 2022 and December 2024. An initial diagnosis of OVF was established when all of the following criteria were met: (1) age > 60 years and lumbar bone mineral density T‐score ≤ −2.5; (2) low‐energy trauma or no identifiable traumatic event; (3) absence of fever or systemic inflammatory symptoms at presentation; and (4) MRI findings consistent with a benign OVF, including vertebral bone marrow edema without evidence of discitis, pedicle or posterior element involvement, epidural mass, or paravertebral abscess.

A final diagnosis of nonosteoporotic pathological vertebral fracture was established when an alternative etiology was confirmed by at least one of the following reference standards: (1) histopathological evidence of acute or chronic inflammation, granulomatous inflammation, or malignant plasma cells; (2) microbiological evidence, including positive blood culture, positive acid‐fast staining, or positive disease‐specific serological testing (e.g., T‐SPOT or Rose Bengal plate agglutination test (RBPT)); or (3) hematological evidence of monoclonal protein identified by serum and/or urine immunofixation electrophoresis.

## 3. Case Descriptions

### 3.1. Case 1

#### 3.1.1. Patient Information

A 70‐year‐old woman presented with low back pain and restricted mobility for 7 days without identifiable cause. No history of trauma or fever. Lumbar bone mineral density T‐score: −3.5.

#### 3.1.2. Clinical Findings

Physical examination revealed tenderness over the L3 vertebra and restricted lumbar mobility, with no neurological deficits.

#### 3.1.3. Diagnostic Evaluation

**Figure 1:**
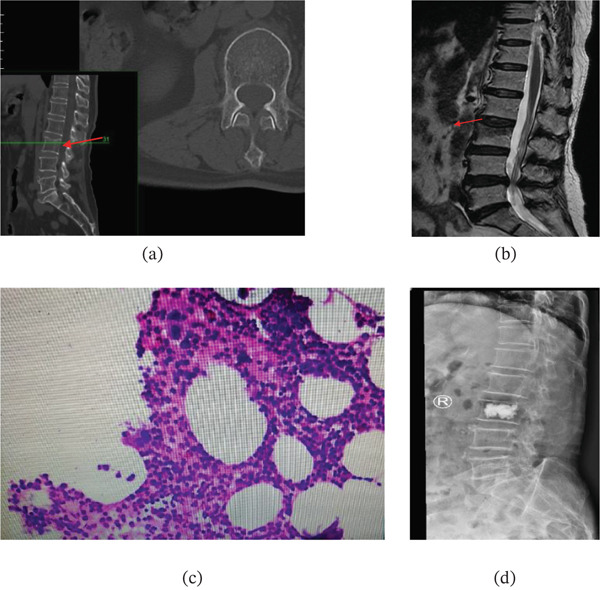
Imaging and pathology of the patient in Case 1 (pyogenic spondylitis misdiagnosed as OVF). (a) Preoperative sagittal CT showed no obvious bone destruction at L3. (b) Sagittal T2‐weighted/STIR MRI showed L3 vertebral marrow edema and compression fracture without discitis, epidural extension, or paravertebral soft‐tissue abnormality—findings that contributed to the initial misdiagnosis of a benign OVF. (c) Intraoperative vertebral bone marrow aspiration biopsy during PVP showed suppurative inflammation, confirming pyogenic spondylitis. (d) Postoperative lateral radiograph showed satisfactory cement filling after L3 PVP.

Lumbar MRI suggested an L3 OVF without signal abnormalities in posterior elements (Figure 1a,b). Laboratory tests showed an elevated erythrocyte sedimentation rate (ESR) of 32 mm/h (reference < 20 mm/h); other results were normal. The initial diagnosis was OVF, with the elevated ESR attributed to postfracture inflammation. This interpretation was based on three considerations: (1) the ESR elevation was modest; (2) the patient had no fever, chills, or other systemic symptoms suggestive of infection; and (3) MRI showed no discitis, epidural involvement, or paravertebral soft‐tissue abnormality.

#### 3.1.4. Therapeutic Intervention

Percutaneous vertebroplasty (PVP) was performed for pain relief. Intraoperative vertebral biopsy revealed pyogenic spinal infection (Figure 1c). Postoperative X‐ray confirmed satisfactory cement filling (Figure 1d).

#### 3.1.5. Follow‐up

The patient received 6 weeks of antibiotic therapy based on susceptibility testing, resulting in complete symptom resolution.

#### 3.1.6. Informed Consent

Written informed consent was obtained.

### 3.2. Case 2

#### 3.2.1. Patient Information

An 89‐year‐old woman was admitted with thoracic back pain of 6 months′ duration. No history of trauma or fever. Lumbar bone mineral density T‐score: −4.2.

#### 3.2.2. Clinical Findings

Physical examination revealed tenderness over the T10 vertebra and restricted thoracolumbar mobility, with no neurological deficits.

#### 3.2.3. Diagnostic Evaluation

**Figure 2:**
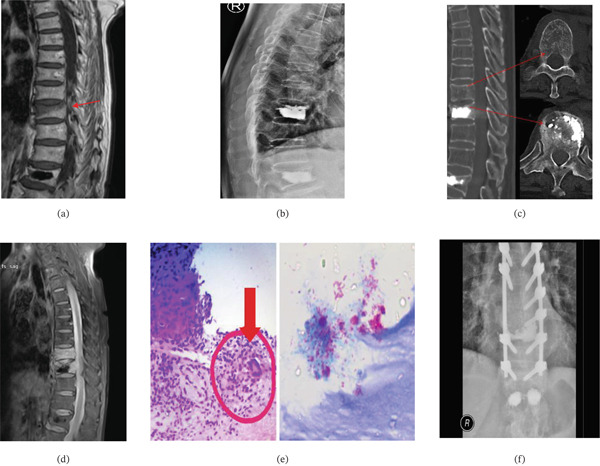
Imaging and pathology of the patient in Case 2 (tuberculous spondylitis misdiagnosed as OVF). (a) Preoperative sagittal MRI showed an isolated T10 vertebral compression fracture without discitis or paravertebral abnormality—leading to the initial interpretation of a benign OVF. (b) Postoperative lateral radiograph demonstrated satisfactory cement filling after T10 PVP. (c–d) 6 months after PVP, CT and MRI showed progressive T9–T10 bone destruction with paravertebral abscess formation, retrospectively suggesting that the original T10 fracture was the initial presentation of tuberculous spondylitis. (e) Preoperative needle biopsy showed granulomas with Langhans giant cells and positive acid‐fast staining, confirming tuberculous spondylitis. (f) Posterior instrumentation and fusion were performed to restore thoracic spine stability.

Thoracic MRI suggested a T10 OVF (Figure 2a). The initial diagnosis was OVF. Following PVP (Figure 2b), the patient was readmitted 6 months later with bilateral costal margin pain. CT and MRI revealed T9–T10 vertebral infection with a paravertebral abscess (Figure 2c,d). Laboratory tests showed ESR 50 mm/h, CRP 25.71 mg/L (reference < 10 mg/L), and a positive T‐SPOT test. Preoperative needle biopsy revealed granulomas with Langhans giant cells and positive acid‐fast staining (Figure 2e).

#### 3.2.4. Therapeutic Intervention

The patient underwent T9–T10 thoracic debridement and posterior instrumented fusion (Figure 2f). Postoperative pathology confirmed tuberculous spondylitis.

#### 3.2.5. Follow‐up

Antitubercular therapy was administered postoperatively. Spinal stability was achieved, and symptoms resolved.

#### 3.2.6. Informed Consent

Written informed consent was obtained.

### 3.3. Case 3

#### 3.3.1. Patient Information

A 74‐year‐old woman was admitted with a 1‐year history of low back pain, which had worsened over the past 2 weeks. She had previously received symptomatic treatment at a local hospital. She denied any history of travel, trauma, or fever. Lumbar bone mineral density T‐score was −3.2.

#### 3.3.2. Clinical Findings

Physical examination revealed localized tenderness over the L4 vertebra and restricted lumbar mobility, with no neurological deficits. Body temperature was normal on admission.

#### 3.3.3. Diagnostic Evaluation

**Figure 3:**
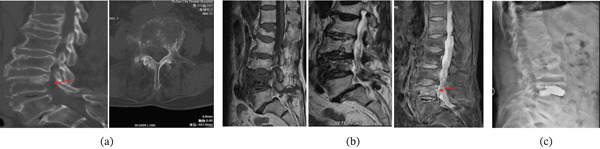
Imaging and clinical course of the patient in Case 3 (brucellar spondylitis misdiagnosed as Kümmell disease). (a) Preoperative sagittal CT showed multilevel vertebral height loss. Axial CT at L4 revealed subtle marginal bone erosion without obvious sequestrum formation—a pattern initially attributed to the nonunion of Kümmell disease rather than infection. (b) MRI showed an intravertebral cleft‐like signal at L4, which further reinforced the erroneous diagnosis of avascular necrosis. (c) Postoperative lateral radiograph after L4 PVP showed satisfactory cement filling. Brucellar spondylitis was later confirmed by positive blood culture for *Brucella melitensis* and a positive Rose Bengal plate test.

CT and MRI demonstrated L4 vertebral destruction with nonunion, consistent with Kümmell disease (Figure 3a,b). Laboratory tests showed an elevated ESR of 50 mm/h (reference < 20 mm/h), whereas C‐reactive protein (CRP) and white blood cell count were within normal ranges. The initial diagnosis was Kümmell disease.

#### 3.3.4. Therapeutic Intervention

PVP was performed at L4, and postoperative imaging confirmed satisfactory cement filling (Figure 3c), with short‐term pain relief achieved. However, on postoperative Day 5, the patient developed fever (maximum temperature 39°C) accompanied by recurrent pain. Repeat laboratory tests revealed significantly elevated inflammatory markers (ESR 62 mm/h, CRP 38.38 mg/L). Spinal infection was highly suspected. Blood culture was performed and yielded *Brucella melitensis*. RBPT) was positive, leading to a final diagnosis of brucellar spondylitis.

#### 3.3.5. Follow‐up

The patient and her family refused surgical debridement. She was treated with doxycycline combined with rifampicin, resulting in gradual symptom relief and normalization of inflammatory markers.

#### 3.3.6. Informed Consent

Written informed consent was obtained.

### 3.4. Case 4

#### 3.4.1. Patient Information

A 70‐year‐old woman was admitted with low back pain that worsened with activity. Lumbar bone mineral density T‐score was −2.9.

#### 3.4.2. Clinical Findings

Physical examination revealed tenderness over the L2 vertebra, with no neurological deficits.

#### 3.4.3. Diagnostic Evaluation

**Figure 4:**
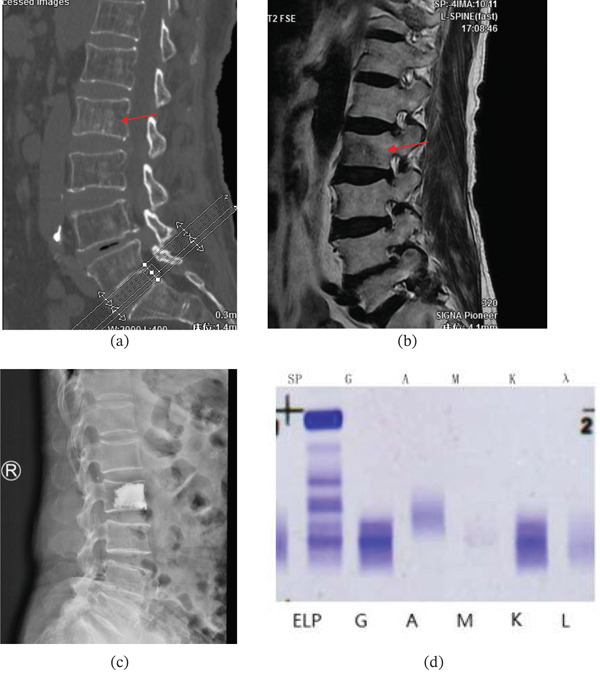
Imaging and laboratory findings of the patient in Case 4 (multiple myeloma misdiagnosed as OVF). (a) Preoperative sagittal CT showed no obvious bone destruction at L2. (b) Sagittal MRI showed signal changes in the L2 vertebral body without focal paravertebral mass or epidural extension—the absence of these malignant features paradoxically reduced suspicion for myeloma, a recognized diagnostic pitfall. (c) Postoperative lateral radiograph after L2 PVP showed satisfactory cement filling. (d) Serum immunofixation electrophoresis revealed an IgG‐*κ* monoclonal protein, confirming multiple myeloma.

CT and MRI indicated an L2 OVF (Figure 4a,b). Laboratory tests showed a decreased albumin/globulin (A/G) ratio of 1.13 (reference range 1.5–2.5). The initial diagnosis was OVF.

#### 3.4.4. Therapeutic Intervention

PVP was performed. Postoperative X‐ray demonstrated satisfactory cement filling (Figure 4c). Immunofixation electrophoresis confirmed the presence of an IgG‐*κ* monoclonal protein (Figure 4d).

#### 3.4.5. Follow‐up

The patient was referred to the Department of Hematology for further management.

**Figure 5:**
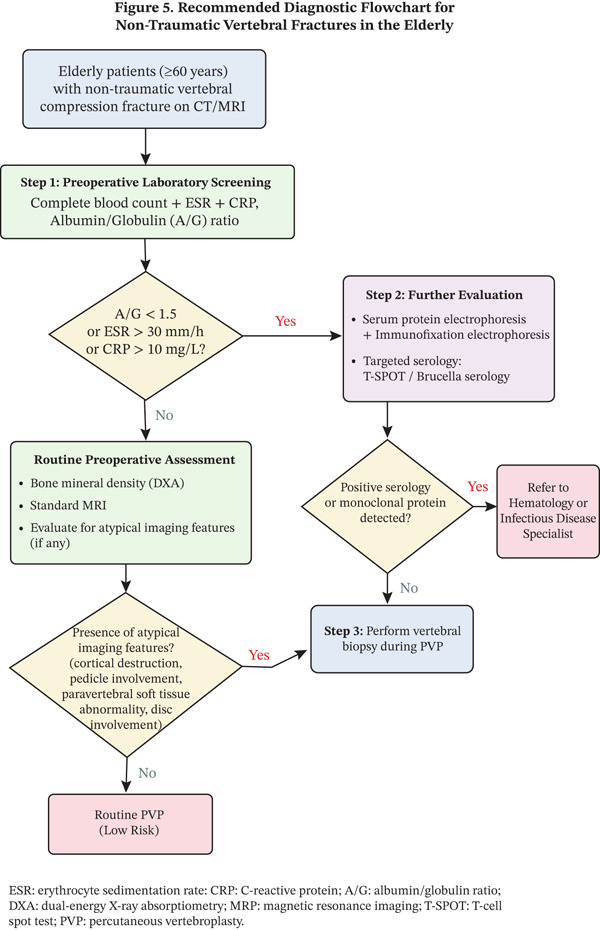
Proposed diagnostic algorithm for the evaluation of elderly patients with suspected osteoporotic vertebral fractures. The algorithm incorporates routine preoperative laboratory screening (ESR, CRP, and A/G ratio), further serological and protein studies when abnormalities are present, imaging assessment, and criteria for selective intraoperative vertebral biopsy during PVP.

As this was a retrospective case series, the specific imaging sequences, laboratory tests, and biopsy methods were determined at the discretion of the treating clinicians according to the initial clinical and radiological impressions. This variability reflects the lack of a universally standardized diagnostic protocol for suspected OVF in real‐world practice and constitutes the clinical gap that prompted the present analysis and the proposed diagnostic algorithm (Figure 5).

## 4. Discussion

### 4.1. Diagnostic Challenges of Pathological Fractures

Pathological vertebral fractures in elderly patients may clinically and radiologically mimic OVFs, leading to misdiagnosis [5–7]. The four cases presented in this series illustrate the breadth of this diagnostic challenge across three etiological categories: acute pyogenic infection (Case 1), chronic granulomatous infection (Cases 2 and 3), and hematological malignancies (Case 4). In each case, the initial diagnosis of OVF appeared reasonable on clinical and radiological grounds, yet proved incorrect. Case 1 demonstrates that pyogenic spondylitis can present with minimal systemic symptoms and only a mildly elevated ESR [8], thereby mimicking an uncomplicated OVF. Case 2 illustrates the insidious course of tuberculous spondylitis, in which the correct diagnosis was delayed by 6 months. Case 3 highlights the diagnostic overlap between Kümmell disease and brucellar spondylitis, as both conditions may present with intravertebral cleft‐like signals on MRI; it also shows that an isolated preoperative ESR elevation, even in the absence of elevated CRP or leukocytosis, may be the only laboratory clue to an underlying infection [9]. Case 4 shows that MM may present with vertebral fractures as the initial manifestation, with the only preoperative abnormality being a decreased A/G ratio. Collectively, these cases underscore a central message: in elderly patients with vertebral fractures, the presence of osteoporosis does not exclude other etiologies, and even subtle laboratory abnormalities warrant careful consideration.

### 4.2. Limitations of Imaging Techniques

Standard MRI sequences, including T1‐weighted, T2‐weighted, and fat‐suppressed images, are the cornerstone of vertebral fracture evaluation; however, their limitations in distinguishing OVFs from pathological fractures are well documented [10]. In our series, the initial MRI findings in all four cases were interpreted as consistent with benign OVFs or, in Case 3, with Kümmell disease, which is an avascular necrosis‐related variant of osteoporotic vertebral collapse. The absence of characteristic signs of infection, such as discitis, paravertebral phlegmon or abscess, and endplate erosion, in Cases 1, 2, and 3, together with the absence of focal masses, pedicle involvement, or epidural extension in Case 4, contributed to the initial misdiagnosis. Advanced MRI techniques may help narrow this diagnostic gap. Diffusion‐weighted imaging (DWI) has demonstrated high sensitivity (86%) and specificity (91%) in differentiating malignant from osteoporotic fractures, because malignant infiltration restricts water diffusion and produces high signal intensity on DWI [11, 12]. In the setting of spinal infection, DWI may also show increased signal within the affected vertebral body and adjacent disc, although this finding is not specific. Chemical‐shift imaging (in‐phase/opposed‐phase MRI) can detect residual microscopic fat within benign fractures, thereby aiding differentiation from malignancy [10]. Although these advanced techniques were not available in our retrospective series, their routine application in cases with atypical features or laboratory abnormalities may facilitate earlier identification of pathological fractures. CT also provides valuable complementary information: cortical destruction, sequestrum formation, or ill‐defined osteolysis should raise suspicion for nonosteoporotic etiologies [13]. In Case 3, the CT finding of vertebral body destruction with nonunion was interpreted as Kümmell disease; however, the same imaging pattern can also be seen in subacute or chronic osteomyelitis, representing an important diagnostic pitfall.

### 4.3. Retrospective Imaging Analysis and Diagnostic Pitfalls

A retrospective review of the initial imaging in our four cases revealed subtle findings that, in hindsight, may have argued against a simple OVF. In Case 1, the marrow edema pattern on MRI was somewhat diffuse and lacked the band‐like subchondral fluid signal occasionally seen in avascular necrosis‐related fractures, yet the preserved adjacent disc signal reinforced the false impression of a benign osteoporotic collapse. In Case 2, the initial T10 compression was isolated and lacked discitis, which was the key reason the lesion was interpreted as an OVF; however, the ongoing severe thoracic pain unrelieved by rest should have prompted closer surveillance. In Case 3, the axial CT demonstrated marginal bone erosion without sequestrum formation, a feature compatible with both Kümmell disease and subacute infection, but the absence of paraspinal soft‐tissue involvement erroneously favored the diagnosis of avascular necrosis. In Case 4, the diffuse and homogeneous marrow signal abnormality without pedicle involvement or an epidural mass did not exclude a malignant lesion, because pedicle involvement is not specific for malignancy and myeloma‐related vertebral fractures may appear benign on MRI [14].

These cases illustrate that conventional CT and MRI findings may be insufficient for differentiating OVFs from pathological fractures when overt infectious or malignant features are absent. In particular, the initial appearance of bone marrow edema, vertebral height loss, and an intravertebral cleft may closely resemble benign osteoporotic collapse, leading to diagnostic anchoring. Importantly, individual MRI signs should not be interpreted in isolation. By contrast, advanced MRI techniques such as chemical‐shift imaging and DWI provide additional diagnostic value, and both have shown useful performance in differentiating benign from malignant vertebral compression fractures. Therefore, when conventional imaging is equivocal or laboratory abnormalities are present, further serological evaluation and selective biopsy should be considered rather than presuming a benign OVF [15].

### 4.4. Role of Laboratory Investigations

Inflammatory markers and serum protein studies provide critical diagnostic clues that may be overlooked when the clinical suspicion for OVF is high. In our series, ESR was elevated in all three infectious cases (Cases 1–3), ranging from 32 to 62 mm/h, yet the degree of elevation alone did not reliably distinguish among etiologies. CRP was normal in Case 3 at initial presentation but rose sharply after symptom exacerbation, illustrating the dynamic nature of this marker in indolent infections such as brucellosis. The total white blood cell count was normal in all four cases, reaffirming its limited utility as a standalone marker for spinal infection [16]. For hematological malignancies, the A/G ratio emerged as a key screening parameter [17]. In Case 4 (A/G ratio 1.13), a decreased A/G ratio was the sole preoperative laboratory abnormality, prompting immunofixation electrophoresis that ultimately established the diagnosis. This pattern is consistent with the pathophysiology of monoclonal gammopathies, in which clonal proliferation of plasma cells or lymphoplasmacytic cells produces excessive monoclonal immunoglobulin, thereby altering the albumin‐to‐globulin balance [18]. We recommend that ESR, CRP, and A/G ratio be routinely included in the preoperative evaluation of elderly patients with suspected OVFs. Serum protein electrophoresis and immunofixation electrophoresis should be performed when the A/G ratio is decreased (< 1.5) or when clinical or radiological features are atypical for OVF. Additionally, specific serological tests (e.g., T‐SPOT for tuberculosis, RBPT, and *Brucella* serology for brucellosis) should be considered guided by epidemiological risk factors [19].

### 4.5. Indications and Methods for Biopsy

Vertebral biopsy should be considered when laboratory abnormalities or atypical imaging features exist. Vertebral bone marrow aspiration biopsy during PVP is a simple and effective diagnostic approach. It adds minimal time and risk to the procedure but significantly improves the detection rate of pathological etiologies. Biopsy is particularly decisive when MRI and laboratory results are discordant. Case 1 (pyogenic infection) diagnosed via this method demonstrates its value in excluding infection. However, the role of routine biopsy during vertebral augmentation remains debated. Some authors do not recommend routine transpedicular biopsy during PVP or PKP [20], whereas a recent systematic review suggests that biopsy may be appropriate when preoperative imaging is atypical or inconclusive [21]. Therefore, we advocate a selective rather than universal biopsy strategy. Specifically, we recommend intraoperative vertebral biopsy when any of the following red flags are present: (1) elevated ESR (> 30 mm/h) or CRP (> 10 mg/L) without an alternative explanation; (2) decreased A/G ratio (< 1.5); (3) atypical imaging features, including cortical destruction, pedicle involvement, paravertebral soft tissue abnormality, or atypical MRI signal characteristics; and (4) discordance between clinical presentation, laboratory findings, and imaging results. It should also be noted that inflammatory markers have inherent limitations. Evidence from infections following vertebroplasty or kyphoplasty indicates that white blood cell count is a poor standalone marker, and although ESR is the most sensitive biomarker, it is not elevated in all patients, even in those with confirmed postoperative vertebral infections [16]. Furthermore, an early‐onset infection after augmentation may represent exacerbation of a preexisting, undiagnosed infection rather than a de novo postoperative complication, and failure to recognize this distinction can be clinically devastating [16]. This reinforces the importance of careful preoperative evaluation, as illustrated by our Case 1, in which the mildly elevated ESR was initially attributed to the fracture itself but later proved to be the only preoperative indicator of an underlying pyogenic infection. Literature supports biopsy use in cases of unexplained fractures [22], although cost‐benefit analysis should be performed to avoid unnecessary invasive procedures [23].

### 4.6. Clinical Recommendations and Future Research Directions

To reduce misdiagnosis, a structured preoperative diagnostic pathway is needed for elderly patients with vertebral fractures. Our case series suggests that this pathway should include: (1) routine ESR, CRP, and A/G ratio testing; (2) serum and/or urine immunofixation electrophoresis when the A/G ratio is decreased or when fracture morphology is atypical; (3) targeted serological studies (e.g., T‐SPOT, *Brucella* serology) guided by epidemiological risk factors; and (4) intraoperative vertebral biopsy when the above‐mentioned red flags are present. A preoperative risk scoring system for “suspected pathological fracture in apparent OVF,” incorporating laboratory parameters, imaging severity, and epidemiological factors, could help identify high‐risk patients earlier. Exploring AI‐assisted diagnostic models to detect subtle nonOVF features on imaging is also a promising future direction [24].

A practical diagnostic algorithm summarizing the recommended approach is provided in Figure 5.

## 5. Conclusion

Pathological vertebral fractures, including pyogenic, tuberculous, and brucellar infections as well as hematological malignancies such as MM, may mimic OVFs in elderly patients and lead to diagnostic delay and inappropriate treatment. Preoperative laboratory investigations, particularly ESR, CRP, and the A/G ratio, provide important early clues to underlying infectious or hematological etiologies. Advanced MRI techniques such as DWI may further improve diagnostic accuracy when imaging findings are atypical. When clinical or laboratory red flags are present, selective intraoperative vertebral bone marrow aspiration biopsy performed during PVP is a safe and diagnostically valuable procedure. A structured, stepwise diagnostic pathway incorporating these elements may help reduce the misdiagnosis rate of pathological vertebral fractures.

## Author Contributions

Jun‐Ming Lin and Xiao‐Jun Yuan contributed equally to this work.

## Funding

This work is supported by the Department of Science and Technology Program of Yichun City, Jiangxi Province, China, JXYC2020KSA003; and Department of Health Commission of Jiangxi Provincial, China, 20177494 202140895.

## Disclosure

During the preparation of this manuscript, AI tools were used solely for language polishing and did not contribute to the scientific content or conclusions.

## Ethics Statement

This case series follows the CARE (CAse REport) guidelines.

## Conflicts of Interest

The authors declare no conflicts of interest.

## Data Availability

Research data are not shared.
